# Toxicological and Biomarker Assessment of Freshwater Zebra Mussels (*Dreissena polymorpha*) Exposed to Nano-Polystyrene

**DOI:** 10.3390/toxics12110774

**Published:** 2024-10-24

**Authors:** Andrew Reynolds, Enya Cody, Michelle Giltrap, Gordon Chambers

**Affiliations:** 1 Radiation and Environmental Science Centre, Physical to Life Sciences Research Hub, Technological University Dublin, City Campus, Camden Row, D02 HW71 Dublin, Ireland; enya.cody@tudublin.ie (E.C.); michelle.giltrap@tudublin.ie (M.G.); 2 School of Food Science and Environmental Health, Technological University Dublin, City Campus, Central Quad, Grangegorman, D07 ADY7 Dublin, Ireland; 3 School of Physics, Clinical and Optometric Science, Technological University Dublin, City Campus, Central Quad, Grangegorman, D07 ADY7 Dublin, Ireland

**Keywords:** nanoparticles, nano-polystyrene, *Dreissena polymorpha*, bioassay, clearance rate, stress biomarker analysis, acute toxicity

## Abstract

The presence of sub-micron-sized plastics in the environment has been increasing, with the possible risks of these particles remaining relatively unknown. In order to assess the toxicity of these particles, 100 nm diameter green fluorescent nano-polystyrene spheres (NPS) (20–60 mg/L) were exposed to zebra mussels (*Dreissena polymorpha*) to investigate the mortality, clearance rate and stress-related biomarker responses. *D. polymorpha* were collected and analysed with standard OECD toxicological tests and biomarker analysis to detect both physical and biochemical responses after exposure to NPS. The toxicity of the NPS to *D. polymorpha* was low, with 60 mg/L NPS causing a mortality rate of 11.1% at 96 h which was statistically significant compared to the 4.2% control. No statistical change could be found for the condition factor (k_c_) of *D. polymorpha* after NPS exposure. Clearance rates in *D. polymorpha* using *R. subcapitata* algae showed NPS-exposed mussels had a reduction of filtering efficiency of up to 30.5%. Bioassay testing shows a mixed but undeniably negative response from the *D. polymorpha* to the NPS, notably a significant rise in DNA Strand Breaks (DSB) and Metallothionein (MT) responses for high NPS concentrations. Additionally, Lipid Peroxidation (LPO) and Ferric Reducing Antioxidant Power (FRAP) assay tests showed a significant increase in response from the higher (>40 mg/L) concentrations of NPS exposure. Although Glutathione S-Transferase (GST) assay showed no statistical change from the control for all NPS-exposed samples, an increase of 20% had occurred for 60 mg/L NPS. Overall, a minimal toxic response from *D. polymorpha* to the NPS exposure below 40 mg/L was seen. After 40 mg/L NPS, mussels presented more acute toxicity in terms of mortality, along with reduced algal clearance rates and anincrease in biomarker response. This study revealed a clear induction of oxidative stress and DSB in the digestive gland of zebra mussels following exposure to nano-polystyrene. While these findings provide valuable insights into the potential harmful effects of nanoplastics in freshwater bivalves, further studies are necessary to help understand the level of threat plastic pollution may pose to the health of freshwater ecosystems.

## 1. Introduction

Across the world, the usage and prevalence of plastic have incurred a vast and growing problem of plastic waste within the environment. Plastics have been disposed of in vast quantities to landfills, burnt, and emitted from large incinerators and discarded into various environments by individuals and industries [1,2,3,4]. Once these plastics have been left within any of these disposal processes, physical or chemical weathering within each environment eventually degrades this plastic into “fragments” that can even reach below submicron sizes [5,6,7,8]. Many of these plastics do not truly degrade and instead produce monomer “clumps” of various sizes, thus maintaining a risk of lingering contamination [9,10,11,12]. In all cases of plastic disposal, micro/nanoplastics (MNP) present an immediate concern to the environment and aquatic species [13]. MNPs are now detected around the world from degrading plastic waste, with various reports and research articles demonstrating the presence of these many forms [14,15,16,17,18]. They have been found in soil and wastewater filtration, spreading to other landmasses and water bodies [19,20,21,22]. MNP contamination is notably high across freshwater bodies and saltwater oceans, as the plastic waste has been washed from landfills into rivers and onto the seas [23,24,25,26]. The toxic effects of MNP in aquatic organisms is an ongoing and intensively studied research topic [27,28,29,30,31,32,33,34,35,36,37]. Prior research outlined that MNPs from plastic fragments or microplastic agglomerates will be ingested by filter feeders, remaining residual within many organs, but primarily the digestive tract [38,39,40,41,42]. Despite prior physiological and spectroscopic experiments on *D. magna* that further demonstrated NPS within the intestinal region, no invasive techniques were conducted, which prevented any biomarker assessments [43]. Additional research displayed a clear risk from the agglomeration/aggregation of plastic particles, indicating the likelihood of riverbed silt contamination along with standard particle suspension [44,45,46,47,48]. As such, analysis was required focusing on the present risks from MNPs entering rivers and lakes on the deep region and bottom-dwelling filter feeders. Zebra mussels (*Dreissena polymorpha*) were chosen to act as a representative of filter-feeding bivalves in freshwater environments [49,50,51,52,53]. These molluscs have become an invasive species in Irish waters (originating in Eastern Europe) and remain relatively resistant to most environments and stresses, allowing them to survive in most conditions. Their capacity to replace existing bivalves and compete for food sources has been so effective in water sources, including Loch Corrib in Co. Galway and Loch Ree in Co. Longford, that the waters clarify from the excessive clearance of freshwater algae and other suspensions in the water [54,55,56]. As a result, these *D. polymorpha* can act as an effective bioindicator as they remain resilient to many chemical components and conditions, remain relatively static in water regions for most of their life, they act as incredibly useful samples to analyse chronic environmental contaminants [57,58,59,60,61]. This enables zebra mussels to become abundant and representative of cultures in determining the chronic effects of MNP across riverbed filter-feeding species using toxicity test models and biomarker bioassays [62,63,64,65,66,67,68]. They are particularly well suited to this task as MNPs have also been detected in wild-caught *D. polymorpha* in lakes around the world [69,70]. They have also been recommended as a bioindicator species for microplastic pollution [71].

Analysis into bivalve mollusc biomarkers has been used extensively in ecotoxicology assessments based on their universal accessibility, thoroughly studied feeding system and relatively extensive life [72,73,74,75,76]. This study implemented standard acute toxicity testing based on OECD No. 121 to initially examine the lethality effects of nano-polystyrene [77]. Additionally, a clearance rate (CR) test and a physical alteration assessment of mass and size, i.e. condition factor (K_c_) were conducted to determine if NPS exposure produced obvious negative impacts on the overall health of the molluscs. Biomarkers of stress responses within the digestive gland (DG) were also conducted to distinguish if short-term NPS exposure could present a toxicological response at a biochemical level, regardless of the acute toxicity tests. Lipid Peroxidation (LPO) is the analysis of malonaldehyde produced to determine fatty acid damage from chemical exposure. The presence of this stress would suggest free-radical chemicals had bound to the unstable NPS surface, causing oxidative stress on the DG it passes through [78]. The DNA Strand Breaks (DSB) biomarker was used to analyse whether the nanoscale NPS had become surface reactive enough to break and damage the DNA within the DG. Metallothionein (MT) is a low-weight protein group responsible for controlling hydroxide radicals produced from oxidative digestion stress and metallic ion contamination. An elevation of MT in the system indicates there is an existing increase in detoxification within the DG [79,80,81]. MT testing was run to determine if hydroxide radicals and/or metallic salts within the media had bound to the NPS and induced oxidative stress. Glutathione S-Transferase activity (GST) release occurs for broad-scale cellular protection from xenobiotics (external chemicals and pollutants) or base peroxidative damage and thus shows whether NPS, or any surface-bound contaminants, induce cellular protein damage on intestinal tissue. Finally, the Ferric Reducing Ability of Plasma (FRAP) assay was used as a broad oxidative stress indicator to the NPS to assist in determining if MT testing results represent oxidative stress or heavy-metal stresses. The combined results of these experiments will allow an evaluation of biological responses in the *D. polymorpha* in relation to NPS particles. This data will add to the body of work demonstrating how producing plastics on the micro/nanoscale provides additional risks and pathways to contamination. This will also directly relate to the need to include the current unregulated micro/nanomaterials [64,82,83,84,85].

## 2. Methodology

### 2.1. Materials and Model Organisms

#### 2.1.1. *Dreissena polymorpha* Collection

Zebra mussels *(D. polymorpha)* were collected from Carrowmoreknock Bay in the lower Loch Corrib region in Co. Galway, Ireland during the month of February. Samples were collected from a boat using a Ponar Grab Sampler to reach underwater rocks (1–2.5 m depth) with attached mussels. Mussels were size checked using Vernier callipers, to ensure only mature *D. polymorpha* of 1–2 cm length were collected. Selected mussels were removed by hand, cleaned and stored in 10.7 L histology buckets filled to 8.5 L with on-site river water. In total, 320 mussels were collected and transported directly to the aquarium in sealed Styrofoam boxes to ensure temperature remain stable during passage.

#### 2.1.2. *D. polymorpha* Maintenance

Synthetic Freshwater (SFW) was prepared for *D. polymorpha* maintenance and exposure treatments as per Smith et al. [86]. A New Brunswick Innova 44R rotatory incubator was set up to run at 30 rpm at 11 ± 1 °C with a day/night cycle of 16/8 h. Once the mussels had reached the lab, the 10.7 L vessels were unsealed and placed into the incubators to acclimatize. *D. polymorpha* were then carefully extracted from the vessel using a small fish net and lightly rinsed and scrubbed with deionised water and a toothbrush to remove any remaining surface debris. They were then placed into two 20 L glass aquarium tanks (160 mussels each) filled with 15 L SFW. *D. polymorpha* were acclimatised for 168 h prior to testing. This process was completed for all wild-caught bivalves prior to testing to ensure depuration and ensure the mussels had acclimatised to the lab-based experimental setup [87,88,89]. SFW was continually aerated to provide sufficient dissolved oxygen and prevent excess ammonia build-up for the mussels. Temperature, pH, algal concentration, and ammonia content of the sample water were tested every other day to ensure ideal maintenance conditions. To avoid any build-up of waste or pathogens, the water in the tanks was 50% replaced twice each week.

Two freshwater algal cultures, *Chlorella vulgaris* and *Raphidocelis subcapitata* were provided by City Analysts Laboratory, Shannon, Ireland. Algal cultures were prepared and sustained in the Radiation and Environmental Science (RESC) building at the FOCAS Institute, TU Dublin. Algae were maintained in a combined New Brunswick INNOVA 44R incubator and shaker in continuous oscillation of 75 rpm and 20 ± 2 °C temperature. The temperature and oscillation were combined with a constant day/night cycle of 16 h/8 h, respectively, operated with both white and photosynthesis light sources to mimic optimal growth conditions [90,91,92]. Algal cultures were suspended in 500 mL Jaworski’s medium and held within 750 mL conical flasks. Every two weeks, the algal cultures were sub-cultured, starting with an algal concentration check using a haemocytometer. Once the concentration was known, a required amount of the stock media was pipetted into new 750 mL conical flasks and diluted to 250,000 cells/mL with 500 mL Jaworski medium to keep the algae healthy. *D. polymorpha* were fed a concentration of ~50 million algal cells (50/50 *R. subcapitata*/ *(C. vulgaris*) into each 10 L of water (200 mL of 250,000 cell/mL stock) twice a week.

#### 2.1.3. Nano-Polystyrene Spheres (NPS)

Thermo Fisher Scientific^TM^ Fluoro-Max G100 polystyrene microspheres (product code: 11868393) of 100 ± 10 nm diameter with integrated Firefli fluorescent green (468/508 nm excitation/emission) dye were used to represent the plastic nanoparticles. The particle’s fluorescence was produced from the styrene chains having divinylbenzene (DVB) dye integrated amongst them, indicating NPS are specially made to produce clear fluorescence without any significant dye leaching effect [93]. These nanoparticles were suspended within deionised water and only contained a small quantity of surfactant (≤0.2 mg/g surfactant to NPS) that was proprietary to Thermo Fisher, and thus, testing could not be conducted. See Supplementary Materials for further comment on the presence of surfactant in NPS products. The concentration of NP in the environment remains largely unknown due to challenges in detection and sampling limitations, primarily because of the extremely small particle sizes. In this study, it was decided to use a concentration range of 20–60 mg/L. This exposure rate would represent the low to medium dose, which would be in keeping with isolated MNP exposures in the environment.

### 2.2. Methods

#### 2.2.1. NPS Toxicological Exposure Testing (NTET)

Experimental set up followed the combination of OECD guidelines for the testing of chemical No. 121 detailed review paper on mollusc life-cycle toxicity testing [77]. This methodology remains under review and incomplete and therefore procedures from OECD 202 (standard for *D. magna*) were altered to suit *D. polymorpha* and maintain an in vivo testing environment [94].

The acclimatised mussels were evenly distributed between twelve test vessels filled with 10 L of SFW that had been oxygenated and acclimatised over 24 h at 11 ± 1 °C. Mussels were divided into control (0 mg/L) and treatment groups. For the treatment groups, *D. polymorpha* were exposed to NPS at concentrations of 20 mg/L, 40 mg/L and 60 mg/L for 96 h. Each vessel contained 24 mussels (1–2 cm in length), which was repeated 3 times for a total of 72 mussels per trial. Control groups were maintained under identical conditions without NPS exposure. The mussels were left to acclimatize for 30 min in the rotatory incubator before concentrated NPS was pipetted into the centre of each vessel at a volume dependent on the final exposure concentration. Each vessel was lightly mixed for 10 s and placed into the rotatory incubator with constant oxygenation and light oscillation (30 rpm). Concentrated NPS was homogenised for 1 min in a sonicator water bath (12 °C) before adding to the test vessel. The toxicity test ran over 96 h. Mussel SFW was not changed during the short 96 h duration of the NTET, with a single NPS exposure at the beginning of the trial. The pH (6.5–7.5) and temperature (9–11 °C) of the test SFW were monitored twice daily. *D. polymorpha* were monitored regularly for deceased mussels to determine variation in lethality from the increased concentration of exposure to NPS over time.

#### 2.2.2. Clearance Rate Testing (CR)

After completion of NTET for *D. polymorpha,* CR analysis was conducted according to the ICES protocol by Widdows and Staff [95]. Mussels were removed and lightly rinsed for 30 s and added to 2 L glass beakers of 1.5 L aerated SFW at 11 ± 1 °C. Ten mussels per beaker were used with three replicates per NPS exposure concentration and the control. *D. polymorpha* were left for 1 h to acclimatise from the transfer. In total, 50,000 cells/mL of *R. subcapitata* were added slowly to each test vessel, returned to the incubator and allowed to homogenise for 5 min before the first sample (1 mL) was taken. Every 30 min over a 3 h period an additional sample was taken from each vessel (72 in total). Algal concentrations were confirmed using a haemocytometer (average across eight subdivisions).

#### 2.2.3. Fulton’s Condition Factor Testing

After the exposure, zebra mussels were examined for Fulton’s Condition Factor (k_c_) (100 * (mass/(length^3^))) to assess physical changes after 96 h testing. Four random mussels per trial were taken and individually measured using Vernier callipers. Once all mussels had been measured for shell length, the sample’s visceral mass was extracted from the shell of the mussel and weighed (wet).

#### 2.2.4. Dissection, Homogenate Preparation for Biomarker Testing

The homogenate production and GST, MT, LPO and DSB tests were conducted based on a selection of biomarker tests already utilized on zebra mussels by Quinn et al. (2011) [96]. An allocated 15 mussels that were not used in the CR analysis were used for bio-marker assay testing. Each individual mussel was wrapped in tinfoil, placed in a sealable plastic bag and transferred to a −80 °C freezer for rapid euthanasia and preservation. During tissue sectioning, mussels were kept at an aquarium temperature of 16 ± 1 °C, on ice and −20 °C cooling pads. Firstly, in order to open the shell of the mussel, a scalpel was used to sever the adductor mussels. The whole visceral mass of the mussels was carefully removed and placed on a pre-chilled (−20 °C overnight) ceramic tile, where the DG was extracted and stored at −12 °C in order to prevent DNA degradation.

The homogenate production was based on the protocols by Quinn et al. (2011) [96]. The DG of the mussel was required for biochemical response testing to the NPS exposure. The DG tissue was removed and homogenized in a buffered HEPES solution (25 mM HEPES sodium hydroxide (HEPES-NaOH), 130 mM sodium chloride (NaCl), 1 mM ethylenediaminetetraacetic acid (EDTA) and 1 mM dithiothreitol (DTT), being diluted in deionised water). Samples were lightly oscillated with a tissue homogeniser until samples were of uniform consistency. A weight (wet): volume ratio of 1 g DG to 5 mL buffered HEPES solution and stored in 1 mL centrifuge tubes. Half of the samples were randomly selected for each NPS exposure concentration and stored at −80 °C freezer for later analysis. This homogenate will be referred to as HOM1. The remaining samples were placed into cooled 1.5 mL polyethylene (PE) Eppendorf tubes but remained in solution at 4 °C, before being centrifuged at 12,000× *g* for 30 min. The DG homogenate supernatant was then separated from the buffered HEPES solution before being frozen at −80 °C, and this homogenate was referred to as HOM2. The remaining tissue containing the rectum, exhalant aperture and gills was not considered usable for current tests.

#### 2.2.5. Glutathione S-Transferase (GST)

Biomarker levels were quantified using a SpectraMax M3 Multi-Mode Microplate Reader throughout this study to measure absorbance and fluorescence intensities for each biomarker described below. Glutathione S-Transferase (GST) activity was examined to determine the levels of GST enzymes in *D. polymorpha* DG after NPS exposure. This method was based on Quinn et al. [96]. Firstly, 50 µL of HOM2 from samples representing all NPS exposure concentrations were put into 8 wells of a 96-well plate along with deionised water. A GST solution was prepared with 250 mL deionised water containing 125 mM NaCl, 10 mM HEPES-NaOH, 1 mM GT prepared in a dark glass bottle placed in a water bath at 35 ± 2 °C. Finally, 1 mM 1-chloro-2,4-dinitrobenzene (CDNB) was quickly added to the GST solution and mixed until all CDNB had dissolved. Next, 0.25 mL of the GST solution was then pipetted into the first 6 wells of each sample in the 96-well plate. The remaining 2 wells acted as a final control to determine that samples free of GST did not produce responses distinct from background emissions under test conditions. The plates absorbance at 340 nm was measured every 5 min up to 30 min. The rate of increase in absorbance was based on the production of protective glutathione and is represented as mM/g (GST activity per glutathione available). The rate of control (0 mg/L NPS) of mussel tissue was then compared to the rates of NPS-exposed samples to determine if the NPS induced an increase in GST production.

#### 2.2.6. Lipid Peroxidation

Lipid Peroxidation (LPO) activity was examined to measure the level of oxidative damage to the lipids of *D. polymorpha* DG after NPS exposure. This test was also based on Quinn et al. [96]. A LPO solution of 150 µL HOM1, 300 µL 10% trichloroacetic acid (TCA), 150 µL 0.67% thiobarbituric acid (TBA) and 1 mM iron sulphate (FeSO_4_) was prepared. This solution was heated to 75 ± 5 °C in a thermo-heater for 10 min before being centrifuged at 10,000× *g* for 10 s, before removing any precipitate from each tube. Samples of 200 µL LPO solution from each NPS exposure concentration were pipetted into separate wells in a transparent 96-well plate. A positive control standard solution was made using 1 mL tetramethoxypropane in 5 mL buffered HEPES solution and added to one well. Fluorescence scans were then run using 516 nm excitation and 600 nm emission to determine the relative concentration of malonaldehyde (μmol/mg) as an indicator for oxidative stress in the DG.

#### 2.2.7. Metallothionein

The methodology based on Quinn et al. [96] was used to determine the levels of metallothionein (MT) in the DG tissue of *D. polymorpha s*amples. First, 0.5 mL of HOM2 was mixed with 0.5 mL 95% ethanol and combined with 8% chloroform in 1.5 mL PE Eppendorf tubes. These solutions were placed into a freezer and brought down to 2 °C, then centrifuged for 10 min at 6000× *g* whilst maintaining the temperature. The formed supernatant was then extracted, and 0.7 mL was pipetted into 1.5 mL Eppendorf tubes and mixed with 1.2 ml 4 °C cooled ethanol, 50 µL ribonucleic acid (RNA) and 10 µL hydrochloric acid (HCL). The MT supernatant was then lightly oscillated before being rapidly cooled to −80 °C in an ultra-low freezer for 25 min, before being brought up to 2 °C and being immediately centrifuged at 6000× *g* for 10 min. All supernatant was removed, and the Eppendorf was filled with a 0.3 mL mixture of 87% ethanol and 1% chloroform. The re-suspended MT supernatant was rapidly cooled to 2 °C in a freezer before being centrifuged again at 6000× *g* for 1 min retained at 2 °C. The supernatant was removed again, with the pellet lightly mixed to re-suspension using 150 µL 0.25 M NaCl, 150 µL 0.2 M HCl with an added 4 mM EDTA. After re-suspension, a 0.3 mL mixture of 2 M NaCl, 0.2 M Tris and 0.4 mM dithionitrobenzoate (Ellman’s Reagent) was pipetted into the tube just before analysis and lightly oscillated. Next, 0.25 mL of each of the final MT solutions were pipetted into separate wells in a 96-well plate. A positive control standard solution of 1 mM glutathione (GT) into a media of 0.5 M HCl, 2 mM EDTA was also added. This was reduced into standards of 250 µL of a range of 0–100 µM GT with 20 µM intervals before the Ellman’s solution was added just prior to analysis. The well plate was then checked for changes in absorbance at 412 nm at 5 and 10 min after Ellman’s addition was measured. The resulting absorption is comparable to nM/mg MT present in the sample.

#### 2.2.8. DNA Strand Breaks (DSB)

DNA Strand Breaks (DSB) were also assessed based on Quinn et al. [96] in *D. polymorpha* DG. Initially, 25 µL HOM1 solution was pipetted into a 1.5 mL PE Eppendorf tubes. A solution of 2% sodium dodecyl sulphate (SDS), 10 mM EDTA, 10 mM tris-hydroxymethyl-aminomethane (Tris) and 40 mM NaOH was prepared and added (200 µL) to the HOM1 solution. The sample was left to homogenise for 1 min before 200 µL of 0.12 M potassium chloride (KCL) was added and heated to 60 °C in a water bath for 10 min. The sample was then inverted several times before transferring to 4 °C for 30 min. The DSB sample was then centrifuged at 8000× *g* for over 5 min at 4 °C. To quantify the intensity emissions from our samples to a µg/mg range, a series of standards were produced from low molecular weight salmon sperm (Sigma-Aldrich product code: 31149) (see Supplementary Materials for further information on standard preparation). Hoechst staining solution was produced by mixing 1 mg/L Hoechst dye, 0.4 M NaCl, 4 mM sodium cholate (Na-Cho) and 0.1 M tris-acetate (Tris-A). Once homogenates and DNA standards were prepared, 50 µL of each media was pipetted into separate wells in a 96-well plate, with three wells per homogenate for NPS exposure concentration or DNA standard concentration. Next, 50 µL buffered HEPES and buffered EDTA solutions was also added to three wells to act as a blank control and a 0 mg/L DNA control, respectively. Once every well was filled, 150 µL Hoechst staining solution was pipetted into every well containing a test media before the plate was gently oscillated for 10 s. The plate was then left for 5 min before fluorescence measurements were run at 360 nm excitation and 450 nm emission.

#### 2.2.9. Ferric Reducing Antioxidant Power (FRAP)

A Ferric Reducing Antioxidant Power (FRAP) assay on the *D. polymorpha* was run with minor alterations to examine the antioxidant capacity of the DG. The FRAP assay demonstrated how efficiently a specific cell plasma is at “reducing” oxidized chemical species that could cause damage to a cell, by showing how well the plasma reduces a controlled concentration of Fe^III^ to Fe^II^ [97,98,99]. This is the only assay independent of the testing system conducted by Quinn et al. It was conducted based on a procedure by Benzie and Strain [98]. The DG of 12 mussels per NPS concentration (20–60 mg/L) and negative control (total = 48) were dissected from mollusc tissue on ice (< 4 °C) before being homogenised into a 1:10 Tris-HCl (pH 7.4). This solution was then placed into several 1.5 mL Eppendorf tubes and centrifuged at 1000× *g* for 15 min at 4 °C. The supernatant was collected, and residual solids were discarded. FRAP Reagent was produced by individually making up the following chemicals and combining them just prior to use. In total, 300 mM acetate buffer (0.775 g sodium acetate with 4 mL glacial acetic acid in 250 mL deionised water), 10 mM TBTZ in 40 mM HCl (0.078 g TPTZ and 1 mL 1 M HCl in 25 mL deionised water) and finally 20 mM iron chloride (0.135 g of FeCl_3_·6H_2_O in 25 mL) were combined at a ratio of 10:1:1, respectively. The volume was dependant on the volume required for final use, as it was utilized by both tissue samples and control runs. The FRAP was mixed in relative darkness to ensure reduced free radical production remained low and allowed for thorough reactivity when added to samples. Calibration used a range of Fe^II^ concentrations from 100 to 500 µM and 1 mM by using FeSO_4_·7H_2_O made up in deionised water (molarity based on full compound, of 278.01 g/mol to calculate molarity). Additionally, 50 µL supernatant or FeSO_4_ standards were combined with 200 µL of the FRAP reagent in each well of a 96-well plate. All samples were then left at 21 °C (room temp.) and immediately scanned to obtain a 0-minute reading. The plate was run under UV–Vis absorbance to check changes in absorbance at 593 nm which directly related to the mM/g FRAP compared to the Fe^II^ range. Following this, the 96-well plates were left to allow antioxidation to occur for 10 min, before the scan was repeated with identical conditions. Darker colour production indicates a higher level of antioxidants within the tissue, and the scan on all tissue should show a rise in mM/g FRAP reading over the 0 to 10-minute scan.

#### 2.2.10. Statistical Analysis

IBM SPSS software V.29 was used for all statistical analysis. All data was of normal distribution. Throughout the acute toxicity testing, CR and biomarker assessments, single factor ANOVA statistical analysis with a probability limit of <0.05 was used to assess if any statistically significant alterations could be found in each trial. When the ANOVA presented statistically distinct responses, *t*-tests were then conducted using the control (0 mg/L NPS exposure concentration) for comparison to the various ranges of NPS-exposed samples to determine where statistically distinct responses occurred. Data are presented as mean ± standard deviation (SD).

## 3. Results

### 3.1. Acute NPS Toxicity

**Figure 1:**
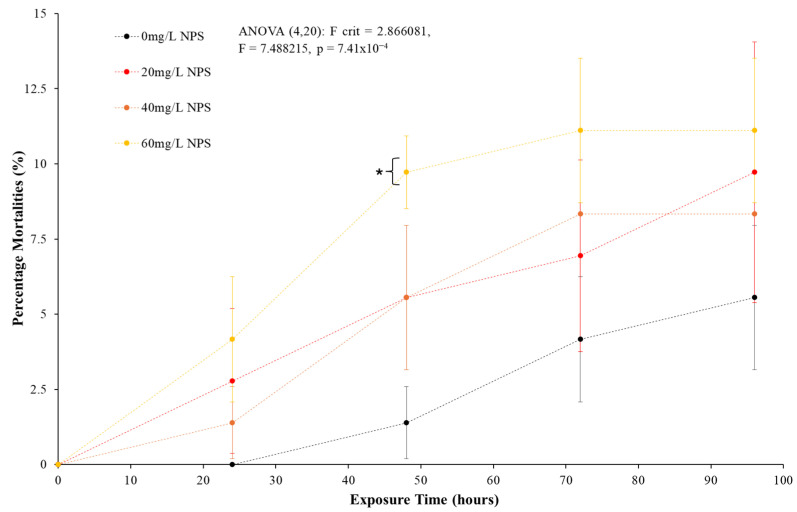
Mean percentage (%) of mortality (±SD) of zebra mussels (*D. polymorpha)* exposed to NPS concentrations at 20 mg/L, 40 mg/L and 60 mg/L for a duration of 96 h (N = 3). Analysis demonstrates distinction in mortality to the NPS presence (including ANOVA validation) with low lethal response across all exposure concentration. * indicates statistical significance at *p* ≤ 0.05 level when compared to the negative control.

The results of the acute toxicity testing for NPS in *D. polymorpha* over a 96 h period found low mortality across all exposure concentrations. Figure 1 presents the results of the exposures, with mussels showing an increase in mortalities compared to the control. Firstly, the control mussels had averaged a 5.6% mortality over the testing, showing mussels remained stressed from the alteration of transport and test containment conditions. By the same timeline, the 20, 40 and 60 mg/L had reached 9.7%, 8.3% and 11.1% mortality, respectively. ANOVA analysis in Figure 1 was conducted over the full spectrum and suggested a statistical distinction in some trials (*p* = < 0.001, F = 7.488, F-crit = 2.866). However, individual *t*-tests showed that the statistical distinction on mortality at any given day of testing varied, with the control (0 mg/L) to 60 mg/L NPS-exposed mussels being mildly statistically distinct at 48 h post-exposure (*p* = 0.037, T = −3.464, T-crit = 2.920) before losing such distinction by 96 h post-exposure (*p* = 0.211, T = −1.000, T-crit = 2.920). The concentration of NPS that was required to cause lethal effects in 50% (LC50) of *D. polymorpha* was not determined. The data indicates that NPS acts as a mild toxin to the mussels, but the source of this toxicity will be validated by bioassay assessment.

### 3.2. Clearance Rate Analysis

**Figure 2:**
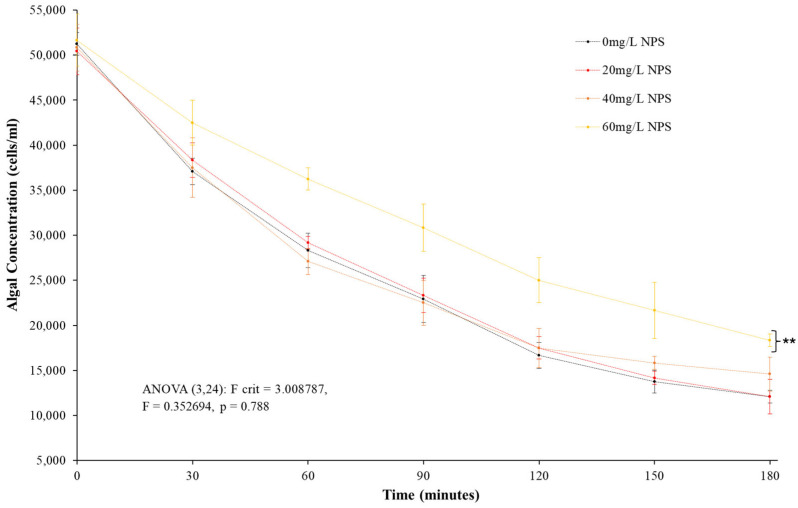
Mean clearance rate (±SD) of *D. polymorpha* of *R. subcapitata* over a 3 h period after exposure to NPS at concentrations of 20 mg/L, 40 mg/L and 60 mg/L for a duration of 96 h (N = 3). ** indicates statistical significance at *p* ≤ 0.01 level when compared to the negative control.

The testing for CR produced a distinct result seen in Figure 2 following 96 h of acute toxicity testing and 3 h of controlled algal clearance. While the control samples and the 20 mg/L and 40 mg/L remained indistinct both in base analysis and statistically, the 60 mg/L samples showed a notable reduction in the rate of algal clearance. Compared to the control samples, the 60 mg/L NPS-exposed *D. polymorpha* had suffered an average reduction of 30.5% in CR. When analysed under ANOVA analysis, the samples do not present clear statistical differences between control samples and NPS-exposed samples (*p* = 0.788, F = 0.353, F-crit = 3.009). However, once a *t*-test compared the control to 60 mg/L, there are clear distinctions in the statistical results compared to the control (0 mg/L NPS) samples (*p* = 0.001, T = −5.941, T-crit = 0.001016). The *D. polymorpha* appear to only demonstrate acute toxicity responses to their CR only after the NPS exposure concentration rises over 40 mg/L. This presents an interesting comparison to the mortality analysis (Figure 1) and suggests NPS have begun entering a stage of acute toxicity by 60 mg/L.

### 3.3. Condition Factor (k_c_) of Zebra Mussels 

**Figure 3:**
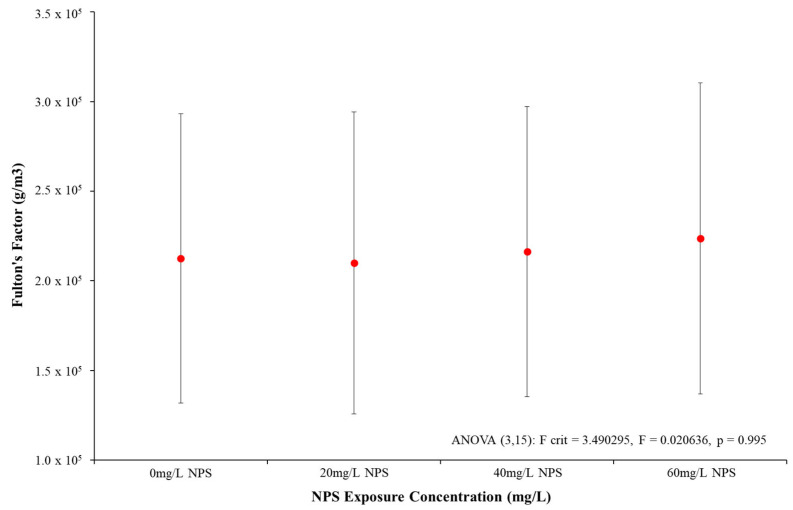
Mean Fulton’s Condition Factor (mass/length^3^) (±SD) of *D. polymorpha* exposed to NPS at concentrations of 20 mg/L, 40 mg/L and 60 mg/L for a duration of 96 h (N = 3).

Examination of the Fulton’s Condition Factor (k_c_) of the mussels over 96 h exposure demonstrated no discernible alteration from control to 60 mg/L NPS-exposed mussels. Figure 3 demonstrated the k_c_ average increased gradually as NPS concentration rose, yet the uncertainties in results severely limited any distinction in k_c_ value from control to NPS-exposed mussels. The results of the ANOVA found no statistically distinct differences in any NPS-exposed mussels when compared to the control (*p* = 0.995, F = 0.353, F-crit = 3.009). These results clearly demonstrated there was no diminishment in key physical dimensions of the mussels over the 96 h of exposure at any NPS exposure concentration. This appears to be validated by the algal cell clearance data (Figure 2), which demonstrated only 60 mg/L exposed would be liable to reduced nutrition, limiting the mussel mass or length.

### 3.4. Biomarker Assay Results

#### 3.4.1. Glutathione S-Transferase Activity

**Figure 4:**
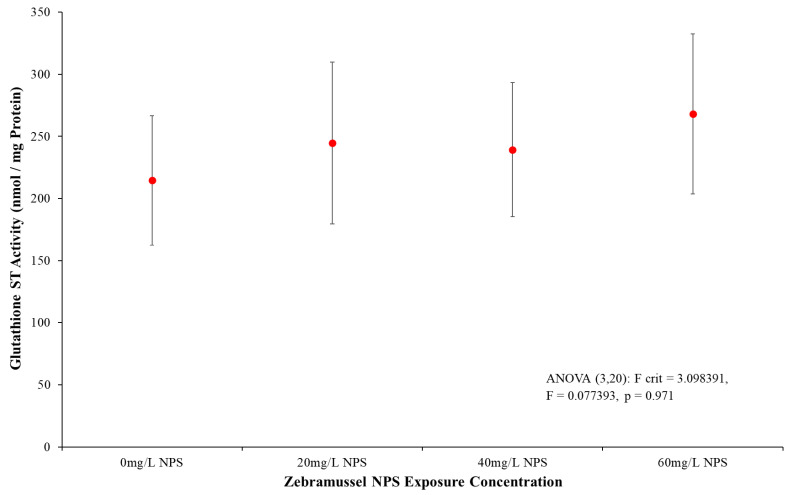
Mean GST (nmol/mg protein) (±SD) for *D. polymorpha* exposed to NPS at concentrations of 20 mg/L, 40 mg/L and 60 mg/L for a duration of 96 h (N = 8). The GST activity of NPS-exposed mussels remained visibly and statistically indistinct from the control data.

The GST activity in *D. polymorpha* DG tissue presented no apparent increase in response from any NPS-exposed samples when compared to the control (Figure 4). By 60 mg/L NPS exposure, the GST activity had only risen on average by 20%, with all samples having significant levels of result variation. ANOVA analysis shown within Figure 4 additionally demonstrated that the GST rates were statistically non-distinct between the control and NPS-exposed samples at every concentration level (*p* = 0.971, F = 0.077, F-crit = 3.098). The slight rise in GST activity averages may potentially indicate some stress as the NPS concentration increased. Further biomarker analysis is warranted to determine whether the NPS would further induce stress on the mussels.

#### 3.4.2. Lipid Peroxidation Activity

**Figure 5:**
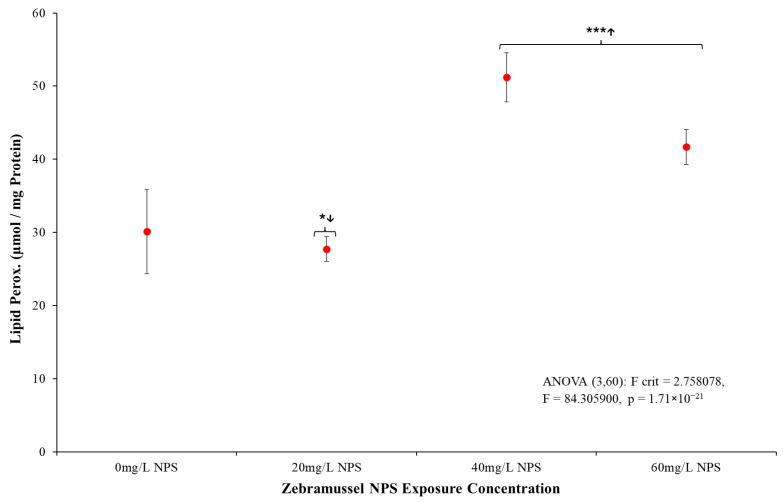
Mean LPO (µmol/mg protein) (±SD) for *D. polymorpha* exposed to NPS at concentrations of 20 mg/L, 40 mg/L and 60 mg/L for a duration of 96 h (N = 12). Mussel tissue for control and 20 mg/L NPS exposure presented no significant discernible sign of LPO stress. *** indicates statistical significance at *p* ≤ 0.001, and * indicates statistical significance at *p* ≤ 0.05 when compared to the negative control. The arrow signifies the direction of the difference (lower/higher) to the control.

Following the GST results, Figure 5 presents a clear rise in *D. polymorpha* LPO activity for individuals exposed to NPS concentrations above 40 mg/L. The ANOVA analysis presented in Figure 5 shows clearly that there was a statistically significant effect to the LPO response after exposure to NPS (*p* = <0.001, F = 84.31, F-crit = 2.758). Once *t*-test analysis was conducted, the lowest level of NPS exposure (20 mg/L) produced an LPO response closely matching the control, yet it was found that 20 mg/L NPS samples had statistically lower LPO response than the control samples (*p* = 0.012, T = 2.842, T-crit = 2.131). However, *t*-tests comparing 40 mg/l NPS (*p* ≤ 0.001, T = −12.136, T-crit = 2.131) and 60 mg/L NPS-exposed mussels (*p* ≤ 0.001, T = −7.762, T-crit = 2.131) both showed NPS-exposed samples had clear statistical increases in LPO response compared to the control. This response is in line with what is shown in the CR data (Figure 2) with mussels showing reduction of filtration rate above 40 mg/L NPS exposure. This also highlights a notable increase in the presence of reactive oxides reacting with DG.

#### 3.4.3. Metallothioneins

**Figure 6:**
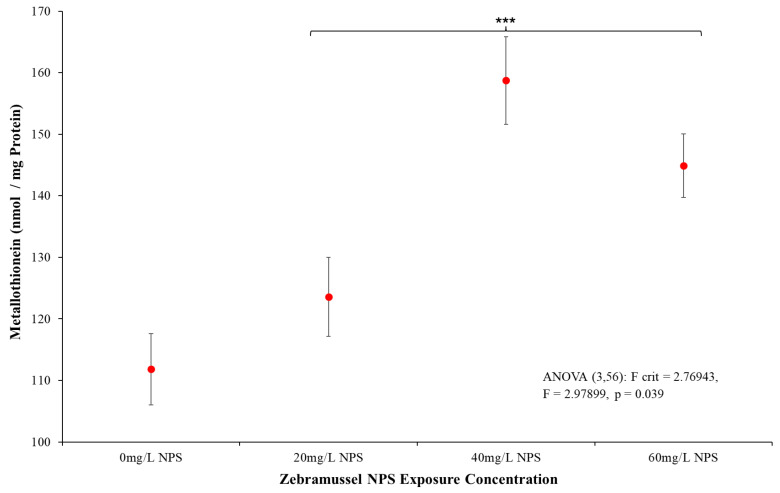
Mean MT (nmol/mg protein) (±SD) for *D. polymorpha* exposed to NPS at concentrations of 20 mg/L, 40 mg/L and 60 mg/L for a duration of 96 h (N = 12). *** indicates statistical significance at *p* ≤ 0.001 level when compared to the negative control.

Following the GST and LPO results, the MT bioassay further complemented and demonstrated that the NPS had a cross-over point of stress response. Figure 6 demonstrates samples exposed to NPS over 40 mg/L presented a statistically distinct ANOVA rise in MT levels (*p* = 0.039, F = 2.979, F-crit = 2.769). Examination of the results also showed control samples (0 mg/L) had a notable larger variation in results to any NPS exposure response. Once analysed under individual *t*-tests, the 20 mg/L samples remained statistically indistinct from the control samples; however, by 40 mg/L exposure, clear statistical differences were presented (*p*-value ≤ 0.001, T = −12.006, T-crit = 2.145). This statistical distinction is also present for the 60 mg/L NPS-exposed samples (*p*-value ≤ 0.001, T = −5.992, T-crit = 2.145), indicating the levels of MT increase were not seen in a dose–response for NPS exposure; instead, the MT seems to have saturation points by 40 mg/L. Similar in response to LPO results (Figure 5), the responses from control samples are low, and 20 mg/L NPS exposure mussels only showed a small and statistically indistinct from the control results. Similarly, MT responses above 40 mg/L increased in the DG tissue. 

#### 3.4.4. DNA Strand Breaks

**Figure 7:**
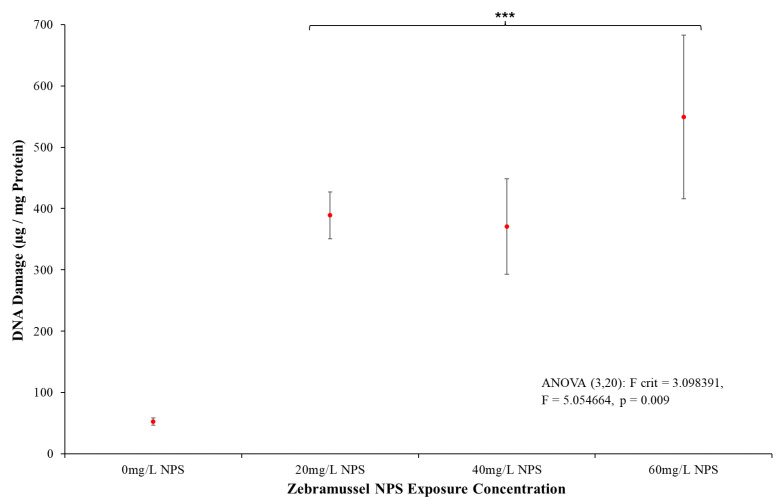
Mean DNA Strand Breaks (DSB) (µg/mg protein) (±SD) for *D. polymorpha* exposed to NPS at concentrations of 20 mg/L, 40 mg/L and 60 mg/L for a duration of 96 h (N = 12). *** indicates statistical significance at *p* ≤ 0.001 level when compared to the negative control.

Upon completing the biomarkers to detect biological responses, DSB was analysed and results are presented in Figure 7. The results indicate that all NPS-exposed samples showed a rise in detected DSB compared to the control (*p* = 0.009, F = 5.055, F-crit = 3.098). *T*-test comparisons found the lowest NPS exposure concentration of 20 mg/L was significantly higher when compared to the control (*p* ≤ 0.001, T = −25.632, T-crit = 2.110). The levels of DNA damage at 20 mg/L showed a 7-fold increase in damaged DNA detected. This statistical difference to the negative control for DSB was also present in the 40 mg/L (*p* ≤ 0.001, T = −5.814, T-crit = 2.110) and 60 mg/L (*p* ≤ 0.001, T = −5.816, T-crit = 2.110) exposed samples. There was no dose–response detected for DSB; however, the degree of variation in results increased with increasing exposure concentration.

#### 3.4.5. Ferric Reducing Antioxidant Power Results

**Figure 8:**
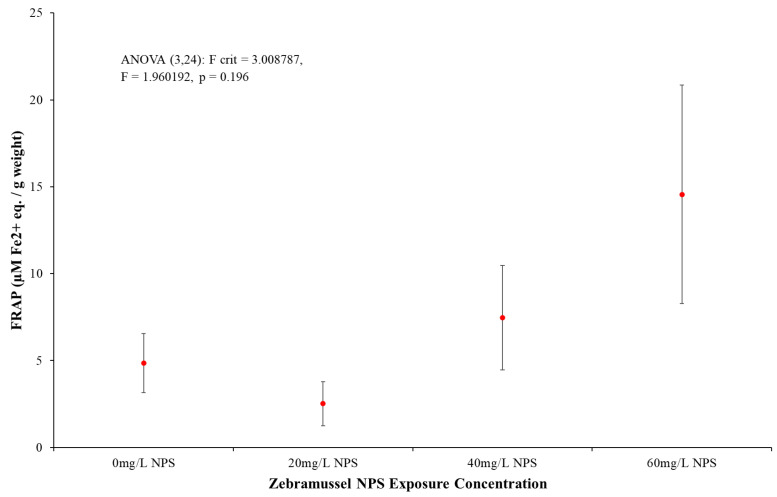
Mean FRAP activity (±SD) for *D. polymorpha* exposed to NPS at concentrations of 20 mg/L, 40 mg/L and 60 mg/L for a duration of 96 h (N = 12).

The FRAP assay results shown in Figure 8 present a similar non dose response of NPS concentration as seen previously in MT and LPO responses. In addition to this, it was clear the degree of variation in higher NPS exposure concentration responses would incur a clear limitation to the results’ average responses (*p* = 0.196, F = 1.960, F-crit = 3.009). In total, 20 mg/L NPS-exposed samples were, on average, lower than the average control FRAP response and were statistically indistinct from *t*-test analysis (*p* = 0.7130, T = 0.386, T-crit = 2.447). Additionally, 40 mg/L NPS-exposed samples produced a minor increase in FRAP response but was not significantly higher than the negative control (*p* = 0.6197, T = −0.523, T-crit = 2.447). By 60 mg/L NPS exposure, there was an increase in average response compared to control results, but the degree of variation was large, leading to a non-significant *t*-test result (*p* = 0.2111, T = −1.400, T-crit = 2.447). Overall, the average responses of the FRAP assay initially suggest an increase in response to an increased presence of NPS; however, statistical analysis clearly indicated this response was not statistically significant compared to control samples.

## 4. Discussion

After reviewing the physical and toxicity responses of *D. polymorpha* to NPS, negative responses but not acute toxicity are demonstrated, with the key acute toxicity test shown in Figure 1 having the highest mortality of mussels (60 mg/L NPS exposure) only reaching an average of 11.1% after 96 h. The statistical difference of NPS to the control samples (average 4.2% mortality) was not shown, with ANOVA analysis indicating statistical significance in mortalities but t-testing showing 96 h results being statistically not significant. Mortalities also remained well below determination of an LC50. The low mortality levels in general from all the NPS exposure concentrations suggest the concentrations of nano-polystyrene incur only a minor and statistically unclear toxic response. The k_c_ measurement has been used to correlate the physical development of aquatic organisms to conditional changes in their environment [100,101,102,103,104,105,106]. The Fulton’s Condition Factor assessment in Figure 2 shows that there was a difference, albeit not significant difference in mass/volume between control and NPS-exposed mussels. For the algal CR (Figure 3) it was clear that there were some effects t at the very least to the 60 mg/L NPS-exposed mussels. The reduction in the rate of clearance of 30% was statistically different from the control analysis, and as such, there could potentially be a risk posed to the mussels. Comparing this to existing models for acute toxicity assessment on bivalves, the NPS appeared relatively non-toxic, particularly at concentrations below 40 mg/L [94,107]. The mussels have the capability to continue the uptake of their food source, and for short periods, appear able to avoid clear impacts on this rate if NPS levels remain below the 40 mg/L threshold. However, when examining and comparing the analysis of CR and toxicity results (Figure 1 and Figure 2), there was a clear concern for mussels exhibiting notable non-linear increases in mortalities and a reduction in algal uptake by 60 mg/L NPS exposure. The possibility behind the minor presence of negative physical responses to NPS might relate to the fact that exposures were not long enough to cause effects relating to common physical indexes (body condition or CR). The NPS might induce a certain amount of toxic response which had not reduced the mussel nutrient uptake over a substantial period, so the visceral mass would not be diminished to notably impact body condition over 96 h. The combination of these three assessment results was useful not just in showing the NPS was not a distinct acute toxin; the three physical assessments additionally provided indications of possible chronic damage from the presence of NPS contamination.

The biomarkers were the portion of the testing run to determine what toxic impacts the NPS were inducing, seen via stress responses in the mussel DG that could incur chronic harm. Glutathione S-Transferase (GST) is an amino-acid isoenzyme released within liver/stomach cells to prevent toxicity from a variety of xenobiotics. This biomarker is a broadly utilized biological antioxidant enzyme required to both prevent and permit repair of cells that have been damaged by free radicals, peroxides and heavy metals [108,109,110,111,112]. GST will interact and catalyse with reactive metabolites on many xenobiotics, eliminating the xenobiotic’s possible reactive pathway that would otherwise potentially react with DNA or cellular proteins, causing damage [113,114]. As the nano-polystyrene spheres interact with the DG tissue, any surface-bound oxidizers would interact with the cells and induce an increased GST activity. From Figure 4, the results were indistinct both on averages and from the statistical analysis. This initially suggested the presence of NPS, even up to 60 mg/L, was not inducing any noticeable response from this common stress biomarker. This did not seem to match even the initial toxicological findings that indicated some level of stress response should have been seen within the highest level of NPS exposure concentration, along with later coming into inconsistency with later bioassay results. One possible reason for the inconsistency in GST response, despite NPS exposure, was that the mussels were undergoing stress separate from their NPS contamination, which altered all responses. There was potential for the acclimatization period to have not been long enough to sufficiently remove existing chemical oxidants from the mussel’s system, or potentially the mussels simply remained unaccustomed and stressed to the new laboratory environment. However, a second and more thoroughly analysed solution was discerned in the works of Quinn et al. [96]. They state that GST activation and oxidative reduction processes from their investigation, along with other researchers, occurred within the first few hours or days from the presence of a contaminant. Assessments carried out at 96 h post-exposure of the GST activity saw only the tail end of the GST response with stressed samples having returned to control levels, the GST process having diminished as much oxidative material as possible. Therefore, in our research, GST antioxidation processes could have occurred and concluded prior to biomarker testing, which reduced the broad oxidation stress as far as possible but failed to prevent all stressors and oxidations from occurring. This would be seen in the later stressor tests, such as LPO and DSB assays, that still present indications of damage the GST activation could not prevent.

The analysis of LPO testing seen in Figure 5 presented a different response to the NPS. LPO biomarker responses from the presence of oxidative free radicals in the DG were statistically distinct in samples above 40 mg/L, inducing damage in the lipid membranes from the NPS. The presence of this stress could suggest free-radical chemicals had bound to the unstable NPS surface, causing oxidative stress on the intestine it passes through [78]. A potential source of the LPO from the NPS was agglomeration, as the NPS were likely exposed to contaminants from the media, algae, or mussel debris. These destabilised plastics that agglomerate and surface-absorbed contaminations have been seen to occur in numerous other studies [115,116,117,118,119,120,121]. Should these contaminants contain reactive species, the agglomerate NPS already present on the DG from ingestion could concentrate the contaminants and induce peroxidation damage [122,123,124]. Compared to the GST results, this remains an unclear source of issue, as oxidative damage to lipids can be incurred from a variety of sources (heavy metals, free radicals, temperature, etc.) [125,126,127,128,129]. However, as samples were contained within controlled media and conditions, certain causes can be disregarded due to low probability as heavy metals were specifically removed from the media and no UV light was present in the room to induce excitation. The MT oxidative biomarker was thus used to determine if the stresses seen in >40 mg/L were consistent in a more specific oxidative stress source. The MT rates shown in Figure 6 demonstrated the DG were undergoing an oxidation stress that was distinct to the NPS exposure and matched the LPO data. MT and LPO are known to directly relate to each other, and so the matching response format (>40 mg/L response) adds to the validation of both assays [127,130,131,132]. As such, the results for both MT and LPO present the primary source of stress in mussel DG induced by either heavy metal or reactive oxide species that have been induced on the NPS surface. Since the media, food source and environment are controlled with the mussels undergoing a 7-day purging period, the likelihood of the issue being heavy metals is incredibly low. As such, the combined GST, MT and LPO test results all show the mussels appear generally uncomfortable in the limited testing environment, but the NPS were clearly either directly or surface-absorbing oxidative species.

To examine how these stresses impacted the DG, the DSB biomarker assay was conducted as a process to analyse cell death from the presence of NPS in increasing concentrations. The detection of DSB is a regularly used indicator of general cell damage and death, with the additional potential of indicating a substance incurs a mutagenic effect [133,134,135,136,137,138]. As such, the results more directly focused on the chronic problem of DG slowly degrading from continual cell death that was not apparent from the acute toxicity assessments. Figure 7 clearly demonstrates even the lowest concentrations of NPS incurred a dramatic increase in DSB compared to the control samples. Polystyrene is not a known cytotoxin, and the media and environment were controlled for heavy metals. The levels of DNA damage make it clear that regardless of the source of the damage, the capacity of the NPS to inflict DSB (and thus likely cell death) was significant. From the response from the DNA damage assay combined with the LPO and MT stress response, evidence was clearly being produced indicating NPS was incurring a detectable effect on the zebra mussels likely to manifest into a chronic toxicity. The already discussed potential for MNP (including the NPS) to become surface reactive and become coated with free radicals from media chemicals not only damage overall cell structure but also degrades core cell organelles [139,140,141]. Whether from the collapse of an overall cell that leaves DNA to degrade from exposure or from the NPS actively breaking the DNA strands from surface reactivity, examining DNA damage is a crucial step in determining if the particle could, over time, induce chronic toxicity or malignant deformities. This could indicate the NPS would become either toxic or chronically mutagenic as the DNA becomes damaged and corrupted.

The FRAP assay was used to determine the tissue plasma’s capacity to respond effectively to a broad range of oxidizing or free radical substances. FRAP biomarker responses would show the current plasma tissue response from samples exposed to an oxidative chemical by an increasing emission of plasma antioxidants [142,143,144]. Presented in Figure 8, the slight increase in average FRAP response from 40 mg/L but most distinctly 60 mg/L NPS-exposed samples could indicate that the plasma in the DG had increased production of antioxidative species in attempts to dampen the lingering presence of the NPS. The average results seem to complement the stressor responses in MT and LPO (Figure 5 and Figure 6) and might still provide some evidence to the theory that the NPS had agglomerated and potentially taken on reactive species. Despite these average results, all responses from NPS-exposed samples were hindered by notably large variations in response, and so NPS-exposed mussel responses to FRAP could not be statistically distinguished from control samples. This FRAP assay was higher than the GST biomarker response (Figure 4) and, taken on its own, the FRAP assay results cannot justify the concept of increased oxidative stress from the presence of NPS. However, considering the results from LPO, MT and DSB, the average value responses from the FRAP assay might still present a relevant response from NPS damage. Had more samples been available, further FRAP analysis on samples would have been conducted to attempt some reduction in result variation.

Overall, evaluating the exposure of NPS on *D. polymorpha* demonstrates only very mild toxicity responses from the standard acute response tests such as mortality, Fulton’s Condition or CR. However, as biomarker assay tests were also conducted to discern more precise indicators of stress or non-lethal damage, more distinct signs of potential damage to the mussels were identified. Control samples and 20 mg/L NPS-exposed *D. polymorpha* showed no clear toxicity or stress responses in most of both physiological and bioassay tests (apart from DNA damage). By 40 mg/L NPS exposure, the mussels begin to demonstrate a statistically distinguished rise in biomarkers for MT and LPO, along with a notable rise in DSB. These results were indicators of the mussels undergoing NPS-induced stress, but the impact of NPS on all the physiological assessments remained low and statistically insignificant to control results. The uncertainty around the increased average response in mortality and FRAP response could have also been indicators of this stress occurring, however it could not be statistically verified. The clearest presentation of NPS acute induced stress occurred for 60 mg/L exposed mussels, which not only had distinctly demonstrated CR going down by roughly 30% compared to control mussels stressed and average mortalities being notably higher than control samples, although not statistically distinct. In addition, the LPO, MT and DSB had all presented clear responses that were statistically significant in comparison to control samples. FRAP assay responses were elevated, but not significantly; however, the results also remained questionable due to the notable high levels of variance in the results. As such while acute toxicity might not have increased compared to control *D. polymorpha*, algal clearance results and increased responses across many stress assays suggest mussels were entering the acute phase of toxicity by 60 mg/L. The result of this data suggests a transition impact of NPS upon the *D. polymorpha*, where the levels of NPS could be tolerated without causing large-scale mortalities or inhibiting the mussel’s capacity to ingest algae. The key concern with the data was how NPS presented a discernible negative impact seen in inducing non-lethal stresses seen across several important biomarker assays, quite specifically DSB.

While 60 mg/L NPS exposure had begun to demonstrate even the acute toxicity response to the NPS, samples in the 40 mg/L NPS exposure and 20 mg/L were still showing signs of damage and stress on a cellular level, which might incur a more chronic impact. Such lingering contamination of NPS or other possible MNP within the DG of the mussel would continue to induce cellular damage [89,145,146,147,148]. The NPS might also have contaminated the algae and diminished the available food supply while adding only non-nutritional NPS to the mussel’s system [149,150,151]. Our own prior research already showed NPS agglomerates in non-purified media, which adhered to the intestines and induced negative effects on *D. magna* [43]. Recent literature has focused heavily on marine bivalve species for MNP assessments, particularly those from the *Mytilus* genus [32,33,34,152,153,154]. Depending on the polymer type, particle size and specific endpoints measured, varying levels of effects have been reported. The exposure of MPs (0.4–950 µm) to *Mytilus edulis* had no effect on CR. DNA damage also remained unaffected, and a decrease in GST activity in the DG was seen. However, an increase in antioxidant enzymes (superoxide dismutase and catalase) was found [154]. The exposure of NPS (50 nm) has been linked to an increase of LPO in DG and gill tissue, along with genotoxicity [33] and decreased cell viability in extracted haemolymph of *Mytilus* spp. [32]. Unfortunately, only a small number of studies have used *D. polymorpha* in their MP toxicology evaluations, with a wide range of exposure times (6–42 days). Reported effects include altered CR [155] and an increased expression of dopamine [147]. No effect of exposure to MP for energy reserves, oxidative stress [147,155] or DNA damage [147] in *D. polymorpha* has been identified. Further research is needed with a focus on the effects of NP in freshwater bivalves to help rectify the current imbalance in the literature, which disproportionately emphasizes marine species.

Limitations within this study include the small sample size used in the zebra mussel exposure system (NTET), which had a secondary impact on the available DG tissue for biomarker analysis. A need for further research with a larger sample size may be required to support these findings. Furthermore, since *D. polymorpha* were collected from a single sample site, a lack of population diversity within the tested organisms is probable. Unfortunately, since *D. polymorpha* are invasive to Irish waterways, selecting a sample collection site required specificity to where this species is known to reside, which ultimately prevented the inclusion of multiple sites.

Overall, the clear implication of this assessment of the results was that the NPS was a substance that needed to be analysed under other model organisms and chronic assessment techniques to fully evaluate the potential harmful effects it could have on zebra mussels and other bivalves, possibly even at lower exposure concentrations. This study supports the use of biomarkers in environmental stress monitoring assessments. Yet, further studies are also required to help uncover the mechanisms of toxicity in aquatic organisms in order to elucidate the toxic mode of the MNP. Accessing the impact of thermal stress on the toxicity of MNP and how it can influence biomarker analysis is also considered a crucial next step in the ecotoxicological analysis of these environmental contaminants [68].

## 5. Conclusions

Existing analysis on freshwater species has already shown how micro- and nanoplastics can induce negative impacts, but most tests are only concerned with the acute toxicity response. This research makes it clear that acute toxicity testing is not flawed in its application. Instead, supplementing standardized acute toxicity tests with assessments that check intermediate demonstrations of stress or damage to the test organisms is recommended. These combined tests provide the initial proof that materials like NPS have chronic risks that appear relatively non-toxic over acute exposure, directing researchers on which forms of chronic assessments should be carried out to ensure environmental safety. Species at the higher trophic level, such as *D. polymorpha,* exposed to substances like NPS using the common acute toxicity assessments of mortality, condition factor or clearance rate, fail to demonstrate the cellular level stress and damage. Our findings using an OECD acute toxicity test present NPS as relatively non-toxic, failing to induce notable casualties or cause notable reductions in physical proportions and only mildly impacting clearance rates at our highest exposure concentrations. However, with the use of stress biomarker assays, there was a clear induction of oxidative stress and DNA damage being impacted on the *D. polymorpha*’s DG. Should the DG tissue be damaged, as seen from this study and in other research, the risk of increased mortality in freshwater bivalves from nano-polystyrene could be problematic.

## Data Availability

The data presented in this study are available on request from Andrew Reynolds.

## Supplementary Materials

The following supporting information can be downloaded at: https://www.mdpi.com/article/10.3390/toxics12110774/s1, Figure S1: Invasive zebra mussels (*Dreissena polymorpha)* from lower Loch Ree region of the Shannon River. References [156,157,158,159,160,161,162,163,164] are in the Supplementary Materials.
