# Supplementary material for: Toxicological and Biomarker Assessment of Freshwater Zebra Mussels (Dreissena polymorpha) Exposed to Nano-Polystyrene
Source: Toxics. 2024 Oct 24;12(11):774. doi: 10.3390/toxics12110774 (PMC11598157; doi:10.3390/toxics12110774)
Supplement: Supplementary file 1 [file toxics-12-00774-s001.zip › toxics-3243869-supplementary.pdf]

Toxicological and Biomarker Assessment of Freshwater Zebra Mussels (*Dreissena polymorpha*) Exposed to Nano-Polystyrene

Andrew Reynolds [1], Enya Cody[1,2], Michelle Giltrap [2] and Gordon Chambers [3,*]

[1] Radiation and Environmental Science Centre, Physical to Life Sciences Research Hub, Technological University Dublin, Aungier Street, D02 HW71, Ireland; enya.cody@tudublin.ie; michelle.giltrap@TUDublin.ie

[2] School of Food Science and Environmental Health, Technological University Dublin- City Campus, Central Quad, Grangegorman, Dublin, D07 ADY7, Ireland;

[3] School of Physics, Clinical & Optometric Science, Technological University Dublin- City Campus, Central Quad, Grangegorman, Dublin, D07 ADY7, Ireland;

* Correspondence: andrew.reynolds@TUDublin.ie; gordon.chambers@TUDublin.ie Tel.: + 353 86 7369971

1. Supplementary Information:

1.1 Materials and Models

1.1.1 Mussel Sample Collection:

Dreissena polymorpha samples were initially planned for collection from the lower Loch Ree region of the Shannon River. However, the mussels appeared to have suffered catastrophic decline in this region over time, so a new sampling location was confirmed and in Carrowmoreknock bay on the lower Loch Corrib region in Co. Galway, Ireland during February. The wipe-out in Loch Ree was assumed to be due to the chronic effect of this invasive species. *D. polymorpha* had been so effective in the loch that they clarified the water from the excessive consumption of freshwater algae and other particles from the water, losing enough algal density that they starved [156-158]. See figure 1 for an image of *D. polymorpha* from a Shannon River rock sample demonstrating the invasive nature of the species.

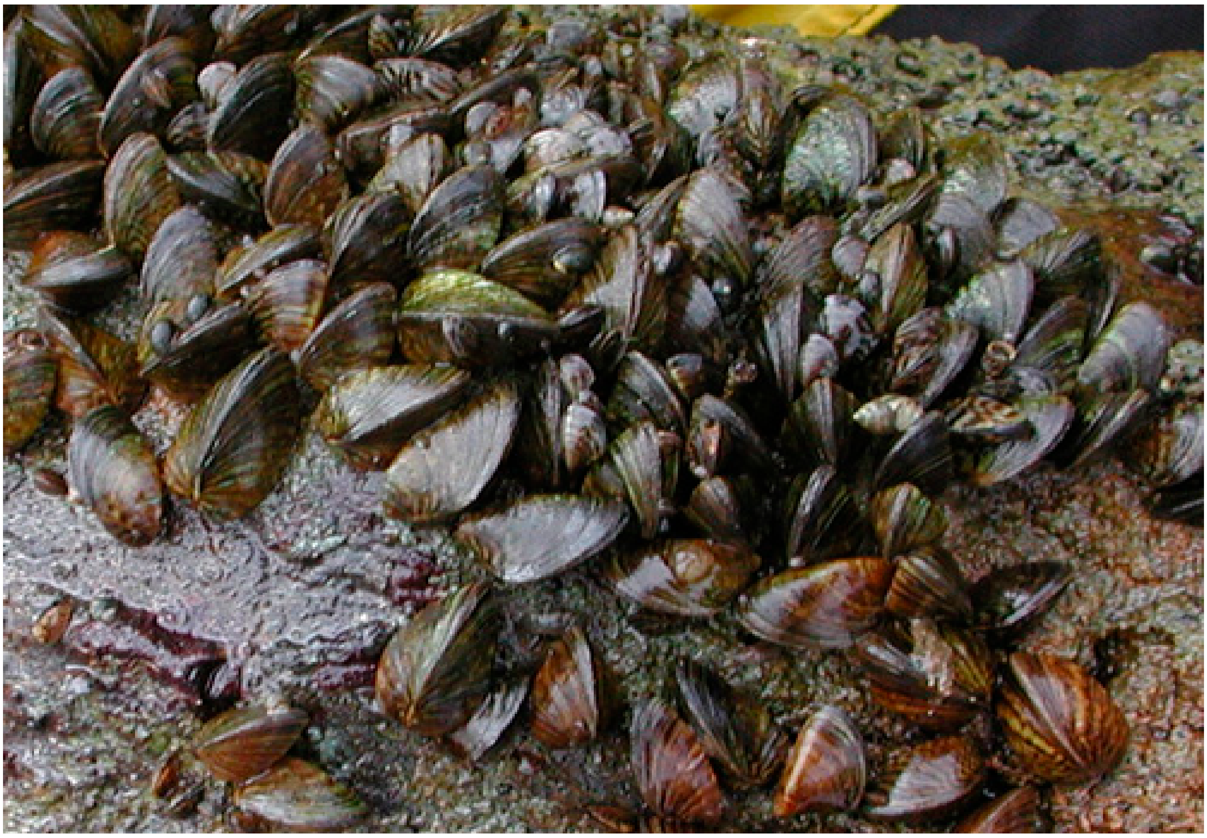

Figure S1 - Zebra mussels (*Dreissena polymorpha*) from lower Loch Ree region of the Shannon River .

1.1.2 Surfactant in NPS Product:

Product Information: Thermo Scientific™ Fluoro-Max Dyed Green Aqueous Fluorescent Particles, 0.10 um polystyrene spheres, 1 % conc. 15 ml.

Product code: 11868393.

Thermo-fisher could not provide MSDS data for the surfactant, but stated the surfactant was similar to sodium dodecyl sulphate (SDS). SDS is a known toxin for aquatic creatures, however there was a distinct lack of literature related to testing SDS specifically to *D. polymorpha*. However, research articles demonstrated molluscs and bivalves of numerous species all presented toxicity 96-hour EC_{50} values in the mg/l range (*C. angulata*- LC_{50} 136 mg/l, *V. nebulosa* - EC_{50} 14.5 mg/L, *H. perovalis* – EC_{50} 6.1 mg/L) [159,160]. Other tests showed that molluscs had a NOEC response to SDS in the high μg/L concentrations (*U. pictorum* - first response 200 μg/L; *P. perna* – 680 μg/L- 48 hours) [161,162]. Other research articles showed the impact of SDS on mollusc functions only becomes notably detrimental in the high μg/ L concentrations, with *M. edulis* shown to only suffering 1.5% loss in clearance rate at 0.5 mg/l SDS [163]. *A. woodiana* having no stress related responses in valve position, shell closure or open-close frequency under 25 mg/l SDS [161]. From our experimental setup the highest NPS exposure concentration was 60mg/l, meaning the concentration of surfactant (≤0.2 mg/g NPS) would be 12 μg/l SDS maximum. When comparing this maximum surfactant concentration is clearly below the most sensitive mollusc responses (EC50 6.1 mg/L or NOEC 200 μg/L) are distinctly below found within the literature examined

and suggests the risk of negative impacts from the surfactant should be minimal. In addition, surfactants are likely to be part of manufactured nano-plastic, their inclusion in the overall toxicity testing without distinction increases the environmentally representative accuracy of the test [164].

1.2 Methods

1.2.1 Algal Clearance Rate: Test Concentration Pre-analysis

Existing literature on the standard clearance/ filtration rates with *D. polymorpha* had a diverse range of responses [156-164]. Due to the lack of specific suspended algal depletion count tests conducted in literature, initial volumes had to be calculated from trial clearance levels given from the *D. polymorpha* tanks to decide an initial algal concentration. During maintenance, a basic test was conducted to find appropriate algal concentrations provide sufficient algae for *D. polymorpha*. This concentration of algae was set within limits to prevent restricted detection from low concentration, but also avoid high concentration that risk a lack of distinguish in algal loss from clearance in different test samples. From these three sources of ideal algal test concentration for *D. polymorpha,* a value of 50,000 cells/ml over a 4-hour testing period.

1.2.2 DNA Damage: DNA Standard Preparation

A DNA standard was produced by first making a stock solution of salmon sperm to a concentration of 1mg/ml. 100ml of buffer solution was mixed and prepared into a 500ml beaker placed into a water-bath at 60°C. The DNA buffer was 50 mM tris-acetate (906 mg in 100 ml) with 1mM EDTA (44.26 mg in 100 ml) to pH 8 in de-ionised water. Once the solution was mixed and at temperature, 100 mg of DNA was mixed lightly into the media until all solid DNA had dissolved into the media. This stock was then diluted into triplicates of 1ml set concentrations using DI water across a range of 0-1000 µg/mg, with intervals of 100 µg/mg and brought down and kept at 4 ºC until use. The salmon sperm standards were prepared on the same day as the testing on mussel tissue occurred.
